# Role of friction on the formation of confined granular structures

**DOI:** 10.1038/s41598-026-39896-4

**Published:** 2026-02-23

**Authors:** Vinícius P. S. Oliveira, Danilo S. Borges, Erick M. Franklin, Jorge Peixinho

**Affiliations:** 1 Faculdade de Engenharia Mecânica, UNICAMP-Universidade Estadual de Campinas, Rua Mendeleyev, 200, Campinas, SP Brazil; 2 Laboratoire PIMM, CNRS, Arts et Métiers Institute of Technology, 151 boulevard de l’Hôpital, Paris, France; 3 Faculty of Physics, University of Duisburg-Essen, 47057 Duisburg, Germany

**Keywords:** Fluidized beds, Liquid-solid transitions, Suspensions, Engineering, Materials science, Physics

## Abstract

Metastable fluidized granular systems can spontaneously defluidize, forming glass- or crystal-like structures. We performed experiments with polymer spheres of different friction and roughness fluidized in a vertical water pipe flow. Velocity fluctuations were higher for high friction materials. Monodisperse particles form a crystal-like shell on the cylinder wall for a range of flow rates and number of particles. For polytetrafluoroethylene (PTFE) spheres with a friction coefficient near 0.1, structural organization was assessed through nearest-neighbor angle analysis, featuring hexagonal packing with defects. At such low friction, defects decreased, while contact chains became longer and more aligned. These findings highlight the role of surface properties in the emergence of ordered or disordered structures, offering new insights into the mechanisms governing glass- and crystal-like arrangements in fluidized particle systems.

Granular materials are ubiquitous on Earth, Earth’s moon, Mars, and other celestial bodies, but their behavior under different conditions is still far from being completely understood, those materials assuming characteristics of either solids, liquids, or gases^1,2^. Besides acquiring a better knowledge on how granular systems behave, granular materials can be studied for understanding problems at smaller scales, such as gas dynamics^3^, interface dynamics^4^ and the formation of resisting structures^5,6^. In the particular case of transitions from liquid-like (dense regime^2^) to solid-like structures, one problem of interest is how and under which conditions either glasses or crystals appear. Jammed or glass-like structures of spherical particles were often considered in numerical simulations using frictionless interactions^7,8^ where the focus was on the search for critical points and scaling laws. When taking into account friction, many contact models explicitly demand a friction coefficient, yet simulations are sometimes performed with an arbitrary friction coefficient. Hence, there is a lack of data of particulate systems where the friction and roughness are well characterized.

One system that can mimic the glass formation process is a solid-liquid fluidized bed (a suspension of solid particles in an ascending liquid) confined in a tube. Very-narrow fluidized beds are of interest because of the effects caused by high confinement, where the behavior of fluidized beds is different from regular (larger) ones. For usual (large) beds with a ratio of tube diameter, *D*, to grain diameter, *d*, in the range $D/d>100$, voids (or bubbles) and slugs usually appear^9–11^. For narrow beds (say $10<D/d<100$), transverse waves, blobs, and voids appear^12^. Yet, for very-narrow beds ($D/d<10$), plugs, crystallization (defluidization), and jamming occur^13–16^. Very-narrow fluidized beds are employed as fluidized-bed bioreactors for biological treatment of domestic wastewater. They are also used in micro fluidized beds (when $D<3$ mm), which are employed in encapsulation^17,18^, pyrolysis^19–22^, gasification^23–25^, and capture of CO$_2$^26,27^. The conclusions obtained for very-narrow beds cannot be extended to larger beds since the bed behavior is highly affected by confining effects.

Goldman and Swinney^28,29^ showed that, when defluidizing (decelerating flow), such system can reach a glass structure (although they did not measure the structure in detail) under water velocities still above that for minimum fluidization, which is the minimum cross-sectional average velocity for fluidization. In this state, they showed that the structure is roughly static, but the grains have some degree of fluctuation. Interestingly, by slightly increasing the water flow from this point, grain fluctuations stop, and the granular structure jams (absence of motion at both the bed and grain scales). In addition, they showed that the formation of this granular glass depends on the deceleration rate of the water (the water flow playing the role of temperature), and proposed that it does not depend on the properties of grains. Later, using a similar system but varying the type of grains, Cúñez and Franklin^15^ showed that bed solidification does not depend on the defluidization rate, but on the particle type (diameter, density, and surface roughness), in opposition to Goldman and Swinney^29^. In addition, Oliveira et al.^30^ showed that under some conditions, solidification can be intermittent, i.e., solid and fluid-like structures can alternate in the system. In the following, we investigate experimentally fluidized beds consisting of different particles to understand how changes in their solid-solid friction properties affect the bed structure.

## Results

The results consist of friction and roughness measurements of the particles, tracking of the particle motion at both large scale and small fluctuations, a phase diagram for the formation of static glass- or crystal-like structures, and the associated timescales. Finally, the static structures are analyzed through angles of contact chains. Note that a dataset containing experimental data and numerical scripts for processing the data are available on an open repository.

### Friction and roughness

**Fig. 1:**
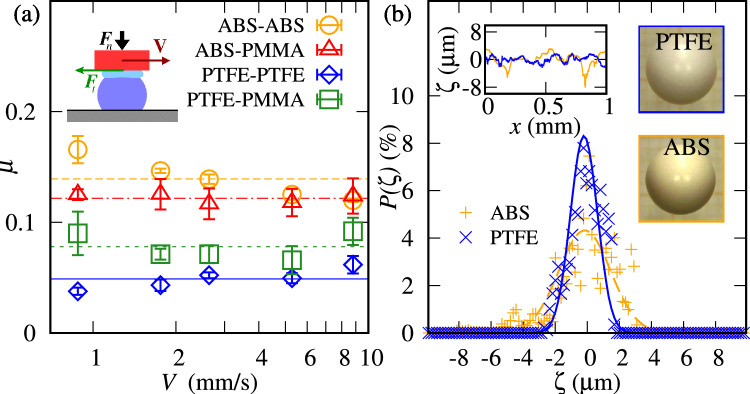
(**a**) Dynamic coefficient of wet sliding friction for the following particle-plate pairs: ABS-ABS, ABS-PMMA, PTFE-PTFE and PTFE-PMMA. The coefficients are plotted as functions of the sliding velocity *V*. Inset, a schematic sketch of the sliding test. (**b**) Histograms of roughness $\zeta$ of spherical particles described by a normal distribution. Insets: profilometer traces of $\zeta$ as a function of distance along the surface, and images of spherical particles: PTFE and ABS.

Figure 1(a) presents the friction coefficient $\mu$ as a function of the velocity *V* of the moving plate for the following couples of wetted spheres and plates, respectively (a drop of water was added between the sphere and the plate): PTFE-PTFE, PTFE-PMMA, ABS-PMMA, and ABS-ABS. The range of velocities is smaller than in the fluidized bed, lower velocity of the plate inducing stick-slip instabilities, whereas larger velocities lead to larger fluctuations for the range of normal forces considered. Overall, a constant dynamic friction $\mu$ is measured and the friction level depends on the particle-plate couple of materials. The lowest value measured was $\mu _\mathrm{PTFE-PTFE}=0.05\pm 0.01$ and the highest $\mu _\mathrm{ABS-ABS}=0.14\pm 0.02$. The relative variation of the friction is merely a factor 3 and can be explained by the roughness of the spheres, which is described by the arithmetic average of roughness height $R_a=\frac{1}{l}\int ^{l}_{0}\left| \zeta \right| dx$, where *l* is the sliding distance of the probe. Table 1 shows also the asphericity and the coefficient of wetted friction between the particle and the wall, the friction measurements being in agreement with previous works^31–33^. We note that the coefficients for wetted friction are within the error bars of those for dry friction (see Table S1 of the Supplementary Information (SI) SI.pdf). For PTFE particles on PMMA tube, $\mu _\mathrm{PTFE-PMMA}>\mu _\mathrm{PTFE-PTFE}$, so the high particle-wall friction seems to promote the crystallization of PTFE particles along the pipe wall^23^.

**Table 1:** Properties of the spheres: diameter, *d*, density, $\rho$, asphericity, average roughness, $R_a$, and measured wetted-dynamic sliding friction between the particle and a wall of the same material, $\mu _{p-p}$ and between the particle and the PMMA wall, $\mu _{p-w}.$

Sphere material	*d* (mm)	$\rho$ (g/cm$^3$)	Asphericity (%)	$R_a$ ($\mu$m)	$\mu _{p-p}$	$\mu _{p-w}$
ABS	$5.91\pm 0.01$	$1.9\pm 0.01$	$0.06\pm 0.02$	$1.25\pm 0.60$	$0.14\pm 0.02$	$0.122\pm 0.004$
PTFE	$5.87\pm 0.01$	$2.33\pm 0.01$	$0.11\pm 0.04$	$0.60\pm 0.21$	$0.05\pm 0.01$	$0.078\pm 0.012$

### Bed motion

**Fig. 2:**
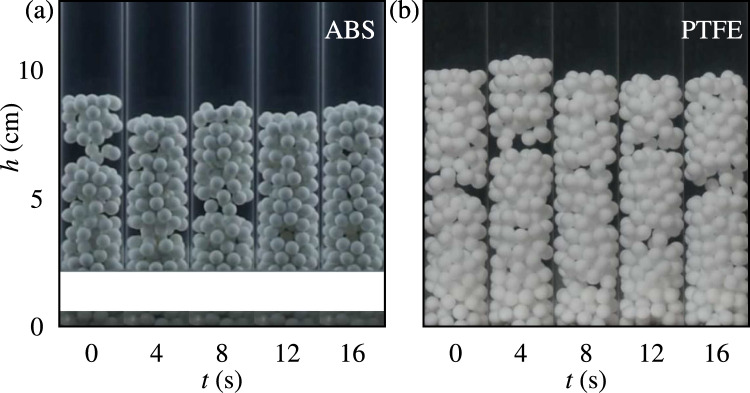
Snapshots of the particle beds consisting of (**a**) ABS particles $N=200$ and (**b**) PTFE particles $N=200$. The white stripe represents an area where data was distorted, hence it is not shown in the figure. The upward water mean velocity is $U=8.7\pm 0.1$ cm/s.

**Fig. 3:**
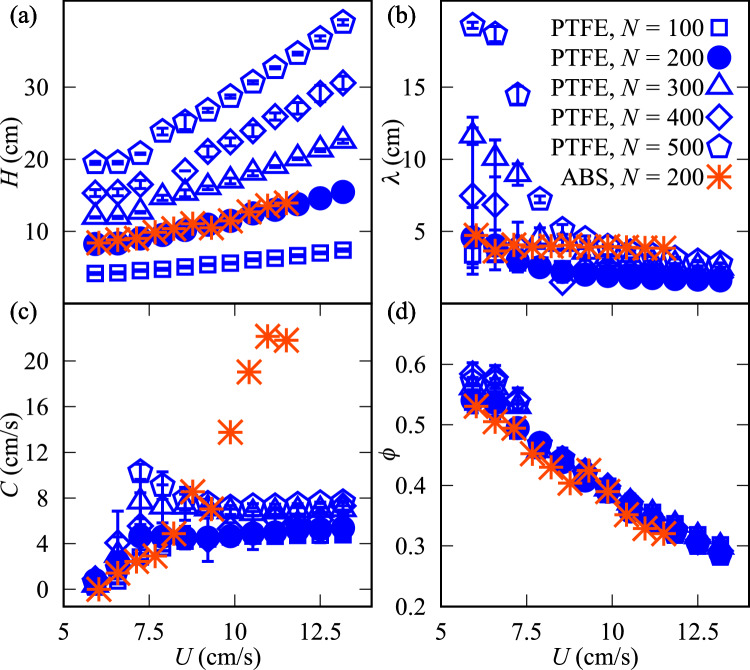
Time averages of properties of beds consisting of *N* particles: (**a**) bed height, *H*, (**b**) plug length, $\lambda$, (**c**) celerity, *C,* and (**d**) global packing concentration, $\phi$, as functions of the upwards water bulk velocity, *U*.

In fluidized beds with $D/d\le 10$, it has been reported that confinement leads to the formation of alternating regions with high and low particle fractions, known as plugs and voids (or bubbles), respectively^13,15^. The presence of force arches within plugs has been reported in previous numerical works^13,14^; however, it is not possible to directly identify such arches from our front-view images. The plugs, characterized by their length, $\lambda$, propagate upward with a typical celerity (velocity), *C*, computed as the difference of the plug position (center of mass) between consecutive frames, divided by the corresponding time interval (Fig. 2).

Figures 3(a-d) show, respectively, the time averages of bed height, *H*, plug length, $\lambda$, plug celerity, *C*, and global volume concentration, $\phi$, as they converge over time. The volume concentration represents the ratio between the volume of the particles and that occupied by the bed as a function of the bulk velocity of the water, *U*, parameterized by the number of particles *N*. *N* varies from 100 to 500 for PTFE, and these beds are compared with that of ABS with $N=200$, extracted from Oliveira et al.^30^. For all beds tested, we observe that their height *H* increases while the plug length $\lambda$ decreases with increasing *U*, $\lambda$ reaching a plateau of approximately 1.6 to 3.6 cm. The increase of *H* with *N* is expected since the size of the system increases (more grains imply a higher bed) and, as the bed expands, the concentration decreases (reflected in the packing fraction in Fig. 3(d)). However, $\lambda$ presents a more complex and non-linear behavior, depending on *U* and *N* (see also fig. S1(b) in the SI). We observe in the experiments that the bed is fluidized in an approximately homogeneous regime when water velocities are just above that for incipient motion, and, by increasing the water velocity, the bed forms smaller plugs. From a certain water velocity on, the size of plugs remains roughly stable with little variation, as we can see in Fig. 3(b). Yet, the same plug-detection algorithm detected some large variability for low velocity cases. By comparing the behaviors of PTFE and ABS particles as a function of *U* (when $N=200$), we notice that *H* follows the same trend and presents the same values, but for $\lambda$ the ABS beds have smoother variations and slightly higher values. The lower values of $\lambda$ with PTFE particles are probably due to their lower coefficient of friction. For the plug celerity *C*, data for PTFE particles show a tendency to reach a plateau, while data do not allow us to conclude the same for the ABS particles (they keep increasing with *U*, and a plateau might possibly appear at high values of *C*).

### Phase diagram

**Fig. 4:**
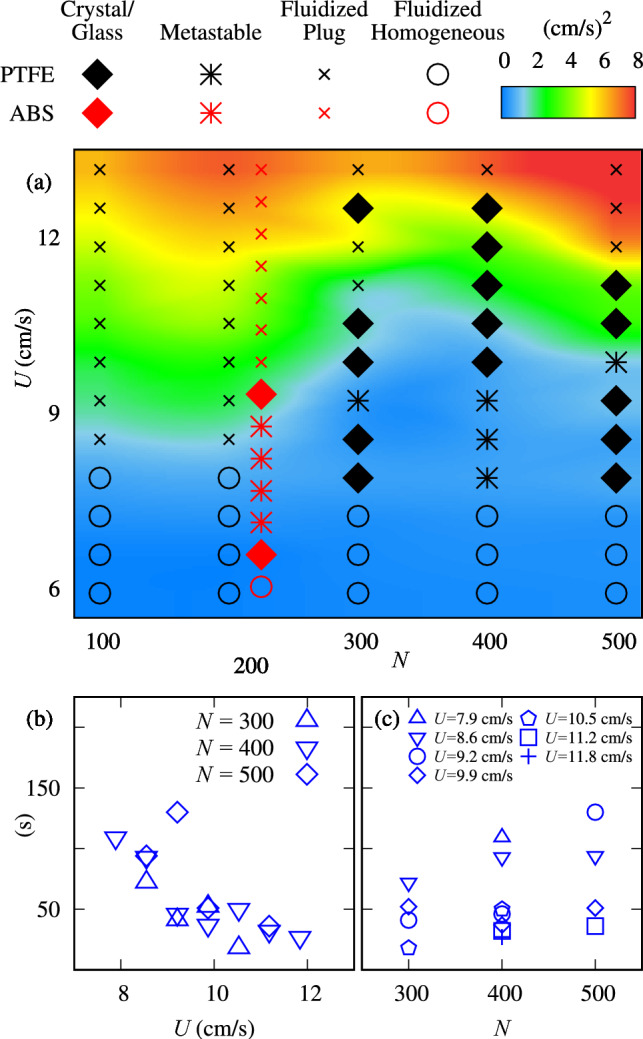
(**a**) Regime map of the time average of ensemble-averaged granular temperature $\overline{\theta }$ in the $N-U$ space, showing the fluidized, crystal/glass, and metastable structures, for the PTFE particles (the regimes for the ABS particles are shown in red symbols). (**b**) Duration of crystal formation, $\tau$, as a function of *U*, for different values of *N*. (**c**) $\tau$ as a function of *N*, parameterized by *U*.

Typically, fluidized beds have an upward flow that exceeds the incipient velocity, transfering momentum from the fluid to solid particles, which in their turn become suspended (see Fig. 3). The suspended particles change their respective positions along time, presenting fluctuations due to the fluid flow and/or interactions with other particles and solid walls. The flow-induced velocity fluctuations decrease with increasing the concentration of particles, and several states are observed: (i) homogeneous fluidized bed, (ii) plug fluidized bed (see Fig. 4), and, in some cases, (iii) no particle motion, which we call here the crystal/glass state. We observed that, depending on *U*, *N* and the type of particles, the bed was either fluidized, formed a static structure (that could be more or less organized, and that we call crystal- and glass-like, respectively), or was in a transient regime that was metastable, where the bed alternatively forms a static structure and refluidizes spontaneously^30^ (see the supplementary video, SV1.mp4, for an example of the formation of the crystal-like structure). As the crystallization/glass transition takes place, it is noticed that the particles form a circumferential layer along the wall, in part because of the lubrication forces close to the wall^34^ whereas the core of the flow remains fluidized for a certain duration.

As we will see in the following, depending on the friction coefficient of particles, the circumferential structure can be more or less organized, reaching a crystal- or glass-like structure. We qualify those structures by making use of Voronoi tessellation and the nearest neighbors’ angles.

Figure 4(a) shows a regime map of time-averaged granular temperatures computed for the ensemble of particles in the $N-U$ space. The ensemble-average granular temperature^2,35^ is defined by equation (1):

1 $$\begin{aligned} \theta (t) = \frac{1}{2M}\sum _{i=1}^{M}\left( {u^\prime }^2_{i}+{v^\prime }^2_{i}\right) , \end{aligned}$$

where $u^\prime _i=u_i-\overline{u}$ and $v^\prime _i=v_i-\overline{v}$ are, respectively, the horizontal and vertical velocity fluctuations of the $i^\textrm{th}$ particle, $u_i$ and $v_i$ being the velocities of each particle *i* and $\overline{u}$ and $\overline{u}$ the corresponding spatial averages, and *M* is the number of analyzed particles. The time average of the ensemble-averaged granular temperature, $\overline{\theta }$, is then computed by Eq. 2,

2 $$\begin{aligned} \overline{\theta } = \frac{1}{T}\sum _{t=0}^{T}\left( \theta \right) , \end{aligned}$$

where *T* is the total duration of the experiment.

**Fig. 5:**
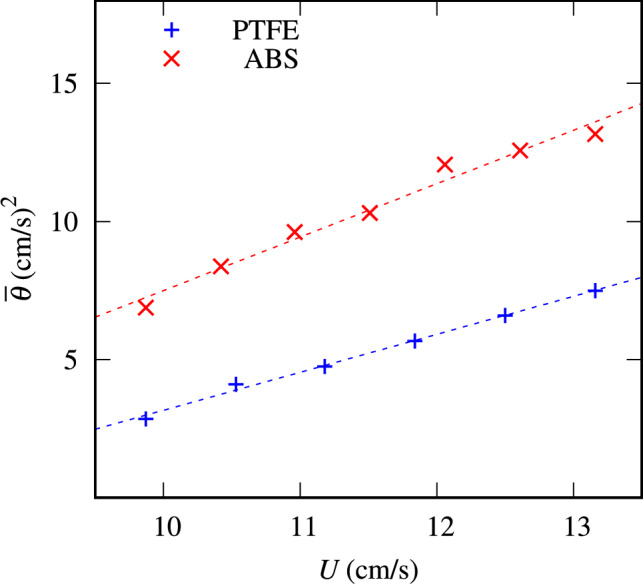
Global granular temperature as a function of the bulk velocity, *U*, in the fluidization regime. The blue points represent PTFE experiments, $N=$ 200, and the red crosses represent the ABS bed with $N=200$. The dashed lines are linear fits. For ABS, the slope is 1.93 cm/s and for ABS, the slope is 1.37 cm/s.

In this map, we distinguish the crystal and glass, fluidized, and metastable regimes, with the fluidized regime presenting two sub-regimes: a homogeneous and a plug fluidization. In the homogeneous sub-regime the bed expands without forming granular plugs (sometimes the bed is only broken into two large portions), and it occurs when water velocities are just above that for incipient motion. The formation of plugs, and then the plug sub-regime, takes place for higher water velocities. In general, for a number of particles $N<300$, the fluidized regime is dominant. As the number of particles increases, from $N=200$ for ABS or $N=300$ for PTFE particles, crystal/glass and metastable states are observed in a range of *U*. The metastable state forms crystal- or glass-like states that are transient (they form and break spontaneously along the duration of the test), whereas the crystal- and glass-like structures are static and steady. If the bed became static in any of the five repetitions, we then marked the point as crystal/glass in the diagram. This is, for example, the case of point $N=300$ and $U=12.5$ cm/s for PTFE, where the bed crystallized in only one repetition. We note that each experiment lasts for 300 s, so that in the crystal- or glass-like states the bed defluidizes before the end of the 300 s and remains defluidized until the end of the experiment. In the metastable state the bed spontaneously alternates between the defluidized and fluidized states along the 300 s. In the regime map, metastable states were observed to coexist with crystal- and glass-like structures at some intermediate *U*.

We measured the time interval, $\tau$, from the start of crystal- or glass-like formation until when the structure reaches its final stable state, and this is shown in fig. 4(b) as a function of *U*, from which we can observe that $\tau$ decreases with *U* (see the supplementary movie SV2.mp4). Figure 4(c) shows $\tau$ as a function of *N* for different values of *U*, and we observe that, as expected, the duration increases with the size of the bed.

In the fluidized regime, the granular temperature increases linearly with *U* (see Fig. 5) and in the range of tested velocities, the average temperatures are higher for the ABS than for the PTFE beds. Using fluctuation-dissipation principles, the energy dissipation from particle-particle and particle-wall interactions is then larger for ABS than for PTFE. Clearly, the vertical component of the temperature should be higher than the horizontal component^36^. Therefore, the ABS beds experience higher temperature gradients during defluidization in comparison with the PTFE beds, leading to the formation of glass-like structures.

### Vonoroi tesselation

**Fig. 6:**
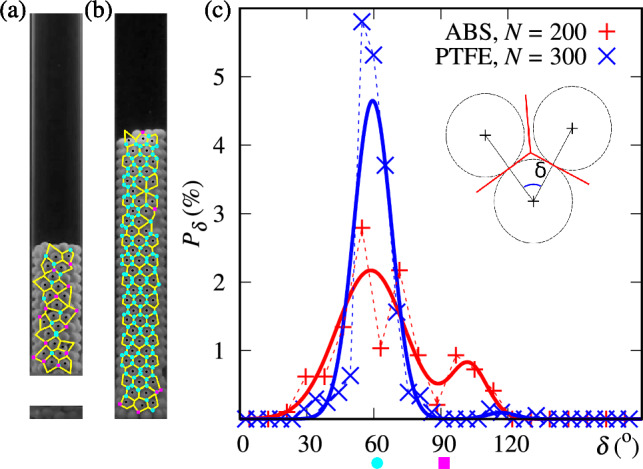
(**a**) Snapshot of a metastable bed superposed with its Voronoi tessellation (in yellow) for ABS with $N=200$ and $U=8$ cm/s and (**b**) snapshot of a crystallized bed superposed with its Voronoi tesselation for PTFE with $N=300$ and $U=8$ cm/s. Cyan points represent the vertices associated with neighbors’ angles of $60\pm 5$°and magenta square points represent the vertices associated with neighbors’ angles of $90\pm 10$°. (**c**) Histogram of distribution of nearest neighbors’ angles $\delta$ for ABS and PTFE crystallized packings. The continuous lines are double Gaussian fits. The insert is a schematic of the nearest neighbors’ angles $\delta$.

The glass- and crystal-like structures form a shell along the pipe wall in our confined system, and, therefore, the position of each particle can be mapped into a 2D cylindrical plane. We now assess the crystal and glass structures using Voronoi tessellation^37^ for which we consider only particle centers that are not at the border of images. We note that the images are two-dimensional, so that some distortions are expected (although assumed not affecting our results). Figures 6(a-b) show examples of Voronoi tesselation (yellow lines) computed for the ABS and PTFE beds, respectively. From that, we can observe a less regular and glass-like structure for the ABS, while the structure is more regular for the PTFE, for this reason we call the latter crystal and the former glass in this work. From the Voronoi cells, the centers and the angles between the nearest neighbors’, $\delta$, can be calculated, and the distribution of $\delta$ is displayed in Fig. 6(c). For crystallized PTFE states, a single peak distribution is obtained and is fitted with the most probable value $\delta =59\pm 7$°. For ABS glass states, two peaks are present and fitted with a double Gaussian model, with $\delta =62\pm 16$°and $108\pm 15$°. For both distributions, the main peak is around $\delta \approx 60$°, which is typical of a hexagonal close-packed lattice, and the second for ABS is $\delta \approx 90$°, which is perhaps related to square packing. Such arrangements are reminiscent of two-dimensional hard-core interactions^38^.

**Fig. 7:**
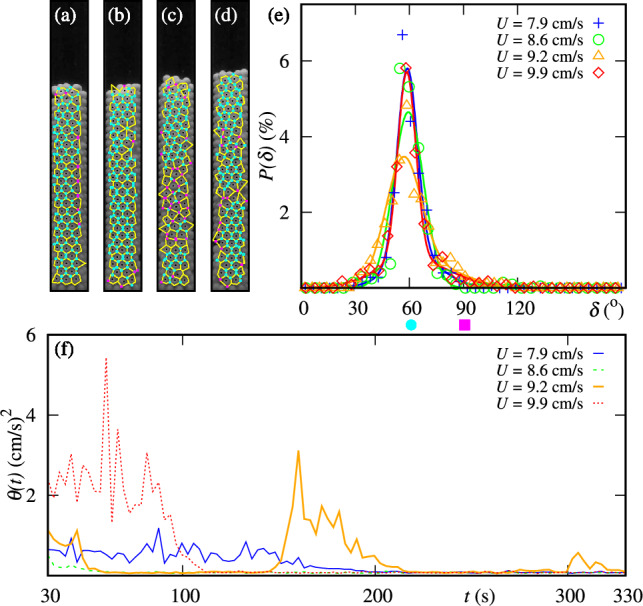
Snapshots of the crystallized bed consisting of $N=300$ PTFE particles and the associated Voronoi diagrams: (**a**) $U=7.9$ cm/s, (**b**) $U=8.6$ cm/s, (**c**) $U=9.2$ cm/s and (**d**) $U=9.9$ cm/s. Cyan points represent the vertices associated with neighbors’ angles of $60\pm 5$°and magenta square points represent the vertices associated with neighbors’ angles of $90\pm 10$°. (**e**) Histograms of nearest neighbors’ angles for different values of *U*. The solid lines represent the fits of the histograms. (**f**) Ensemble-averaged granular temperatures $\theta$ as a function of time *t* for the same cases shown in panels (**a**)-(**e**).

For PTFE, Fig. 7(a-d) present snapshots of the crystal-like structures for $7.9\le U \le 9.9$ cm/s. The tessellation (also represented as yellow lines) and the nearest neighbors’ angles (represented by colored points) of the structure exhibit hexagonal packing. When varying *U*, one can notice that the hexagonal crystallized structure in Fig. 7(c) is discontinuous from bottom to top as indicated by the magenta points (representing $\delta =90\pm 10$°) and it is interesting to note that this discontinuous configuration is associated to a metastable state (see Fig. 4(a)). This evidences the fragility of the structure associated to metastability, not seen in Fig. 7(a), (b) and (d), which form persisting crystal-like structures. In all four cases, the tendency to have a single large peak (see Fig. 7(e)) indicates the formation of hexagonal structures, which are independent of *U*. Finally, Fig. 7(f) shows the time evolution of the ensemble-averaged granular temperatures, $\theta$, of the same four cases, from which we observe that for $U=7.9$, 8.6 and 9.9 cm/s, $\theta$ decreases and remains within values close to zero, while for the metastable case ($U=9.2$ cm/s), $\theta$ alternates between high and low values. Additional evidence of temperature intermittence associated to metastable states for ABS particles are observed (see fig. S2 in the SI).

## Conclusions

In conclusion, fluidization of very-narrow beds consisting of regular particles can lead to fluidization, crystallization/glass formation, or metastable (transient fluidization) regimes, depending on the fluid velocity and number of particles. Within a given range of fluid velocities, the bed is in metastable or crystal/glass regimes, the metastable regime becoming more rare as the fluid velocity is increased, with the crystal/glass formation time decreasing also with *U*. Above this range of *U*, the bed is fluidized. The decrease in the coefficient of sliding friction of particles leads to more organized structures, which we associate with crystals, while those with higher friction coefficients are amorphous, which we associate with glasses. In addition, we show that the (granular) temperature is an important parameter, with high rates of decrease giving origin to amorphous structures, just as it happens for glasses and crystals. Besides fluidization and defluidization, our results represent new insights into the formation of crystals and glasses.

## Methods

### Experimental setup

Here, our strategy was to monitor how the formation of solid structures in confined solid-liquid fluidized beds depends on the properties of the solid particles, specifically the solid-solid friction coefficient, $\mu$. For that, we carried out experiments in a water loop, consisting basically of a water reservoir, a centrifugal pump, a flowmeter, a flow straightener, and a tube of diameter $D=25.4$ mm, which has been used in previous works^15,30^. The test section is a vertical transparent tube made of polymethyl methacrylate (PMMA) mounted just downstream of the flow straightener (a porous media made of 6 mm particles), as depicted in Fig. 2. The fluid was tap water at room temperature, and the resulting flow had a bulk Reynolds number, $Re=\rho _w UD/\eta$, within 1500 and 3300 (typical of transitional pipe flow^39^), where $\rho _w$ is the density of water, *U* is the bulk velocity, and $\eta$ the dynamic water viscosity. Each test was repeated five times to improve statistics.

The fluidized beds consisted of either spherical particles of acrylonitrile butadiene styrene (ABS) or polytetrafluoroethylene (PTFE). The ABS-based particles were used in a previous study^30^. The particles are confined, with the ratio between the tube diameter, *D*, and that of particles, *d*, within 4.2 and 4.3, and their properties are summarized in Table 1. The Stokes number based on the terminal velocity $v_t$ of a single particle, $St=v_t d\rho /(9\eta )$, is 467 and 696 for the ABS and PTFE particles, respectively, showing that they have considerable inertia with respect to the fluid and that particle-particle collisions are not negligible^40^. The average interstitial velocity, defined as $U/\phi$, varies within 10.6 and 44.2 cm/s, where $\phi =2Nd^3/\left( 3HD^2\right)$ is the average packing fraction (global volume concentration of particles), *H* being the time average of the bed height and *N* the number of particles in the bed. The incipient velocity is defined as the bulk velocity at which particles in the bed begin to move^15^ (oscillations of small amplitude detected between frames). We observe that above a minimum incipient velocity, $U_{if}\simeq 4.4$ cm/s for PTFE and $U_{if}\simeq 4.5$ cm/s for ABS, the bed fluidizes, and remains indefinitely fluidized for water velocities above a higher threshold, $U_{max}\simeq 13$ cm/s. Moreover, either crystal- or glass-like formation can occur when $U_{if} \le U \le U_{max}$, and, otherwise, structures in the form of plugs develop with particles migrating from one plug to the other.

### Friction measurements

We measured the friction coefficient $\mu =F_t/F_n$ of the materials involved, where $F_t$ and $F_n$ are, respectively, the tangential (measured) and normal (imposed) forces on a sliding sphere. The dynamic sliding friction measurements were performed using a torsional rheometer Anton Paar MCR 502 with a moving top plate made of PTFE, ABS, or PMMA, and imposing the normal force $F_n$, which can be varied from 0.1 to 5 N. A sketch of the setup is shown in the inset of Fig. 1(a). Because our system is a solid-liquid fluidized bed, we added water at the contact of the sphere with the plate. The tests were performed at room temperature and normal humidity for a duration that varied between 30 and 70 s, the water drop did not evaporate during the test. Previous works^33^ have also investigated PTFE spheres sliding on a substrate, and concluded that the contact of PTFE spheres on dry or liquid-immersed surfaces yield comparable friction coefficients. The bottom plate is drilled so that three spheres of PTFE or ABS are flush-mounted. The measurements consisted in applying $F_n$ and then measuring the torque *T* for a constant velocity *V* of the moving plate. These parameters are tracked during a given time, corresponding to a total sliding distance of about 9 mm. After a short transient, the measurements of $F_n$ and *T* stabilize. $F_t$ was computed as the ratio of the torque, *T*, and the distance *R* from the center of the top plate to that of the contact point with the spheres, $F_t=T/R$, and, afterward, the friction factor $\mu$ was calculated and then averaged for the 20 final time steps. We used particles of ABS or PTFE and plates of ABS, PTFE or PMMA. The coefficient of friction $\mu$ is slightly dependent on *V* within a range comparable to the collision velocities observed in the fluidized beds. This dependence is stronger for the ABS-ABS pair, where the variation can reach approximately 30%. The ABS-ABS case appears to exhibit mixed lubrication behavior as described by the Stribeck curve, while the other cases show an almost constant $\mu$, suggesting a regime of soft tribology over this velocity range^41,42^, which is commonly observed in polymer materials due to their reduced mechanical surface properties.

### Roughness measurements

In addition, the roughness of the particles and plates were measured using a surface profilometer Dektak. Figure 1(b) presents the roughness dispersion of both particles. It is represented as a normal-distribution fit of the measured roughness height, $\zeta$. The surface of PTFE particles exhibits a narrower distribution of $\zeta$ than that of ABS, indicating that PTFE particles are smoother.

### Image analysis

The system is analyzed at both the bed and grain scales by processing sequences of images with resolutions of approximately 100 $\times$960 px, and acquired at 30 Hz for 300 s (more details are available in the supplementary information in Ref^30^.). The field of view was 25.4 $\times$ 244 mm, so that the spatial resolution was approximately 3.9 px/mm. The positions of the particles are tracked and a Voronoi tesselation analysis is carried out in order to extract the distribution of the nearest neighbors’ angles of the particles forming the cylindrical shell with a hollow core. A narrow angle distribution is a signature of a crystal-like structure. Numerical unwrapping of the acquired images introduced distortions in the images that deteriorated the data. For this reason, no calibration for correcting the distortion due to the curvature of the wall was performed, and the particles located at a distance greater than one particle diameter *d* from the wall were overlooked in the Voronoi tesselation.

## Supplementary Information

Supplementary Information 1.

Supplementary Information 2.

Supplementary Information 3.

## Data Availability

The data that support the findings of this study are openly available in Mendeley Data at https://doi.org/doi:10.17632/25ncx7cc42.3.
