# Supplementary material for: Role of friction on the formation of confined granular structures
Source: Sci Rep. 2026 Feb 23;16:7507. doi: 10.1038/s41598-026-39896-4 (PMC12932809; doi:10.1038/s41598-026-39896-4)
Supplement: Supplementary file 1 — Supplementary Information 1. [file 41598_2026_39896_MOESM1_ESM.pdf]

Supplementary Information:
"Role of friction on the formation of confined granular structures"

Vinícius Pereira da S. Oliveira
Faculdade de Engenharia Mecânica, UNICAMP-Universidade Estadual de Campinas, Rua Mendeleyev, 200 Campinas, SP, Brazil and Laboratoire PIMM, CNRS, Arts et Métiers Institute of Technology, Cnam, 151 boulevard de l'Hôpital, Paris, France

Danilo S. Borges
Faculdade de Engenharia Mecânica, UNICAMP-Universidade Estadual de Campinas, Rua Mendeleyev, 200 Campinas, SP, Brazil and Faculty of Physics, University of Duisburg-Essen, 47057 Duisburg, Germany

Erick M. Franklin
Faculdade de Engenharia Mecânica, UNICAMP-Universidade Estadual de Campinas, Rua Mendeleyev, 200 Campinas, SP, Brazil

Jorge Peixinho
Laboratoire PIMM, CNRS, Arts et Métiers Institute of Technology, Cnam, 151 boulevard de l'Hôpital, Paris, France

This supplementary material contains (i) the description of the two supplementary videos, (ii) a table and (iii) two additional figures supporting the main text of the article "Role of friction on the formation of confined granular structures". Fig. S1 presents dimensionless dynamics of the particle beds under varying flow conditions. Table S1 details the physical and frictional properties of the tested spheres. Fig. S2 illustrates local and global granular temperatures and velocity fields for two particle materials. All data complement the experimental analysis discussed in the main manuscript.

Supplementary Video 1 (SV1.pm4). Fluidization and formation of the crystal-like structure with $N = 400$ PTFE particles with bulk upwards water velocity $U = 0.1053$ m/s.

Supplementary Video 2 (SV2.mp4). Fluidization visualization of a metastable state with $N = 300$ ABS particles with bulk velocity $U = 0.1053$ m/s.

Sphere material	d (mm)	ρ (g/cm^3)	Asphericity (%)	R_a (μm)	μ_{p-p}	μ_{p-w}
ABS	5.91 ± 0.01	1.9 ± 0.01	0.06 ± 0.02	1.25 ± 0.60	0.126 ± 0.008	0.122 ± 0.008
PTFE	5.87 ± 0.01	2.33 ± 0.01	0.11 ± 0.04	0.60 ± 0.21	0.057 ± 0.007	0.090 ± 0.010

Table S1. Properties of the spheres: the diameter, d, the density, ρ, the asphericity, the average roughness, R_a, and the measured dry dynamic sliding friction between the particle and a wall of the same material, μ_{p-p} and between the particle material and the PMMA wall, μ_{p-w}

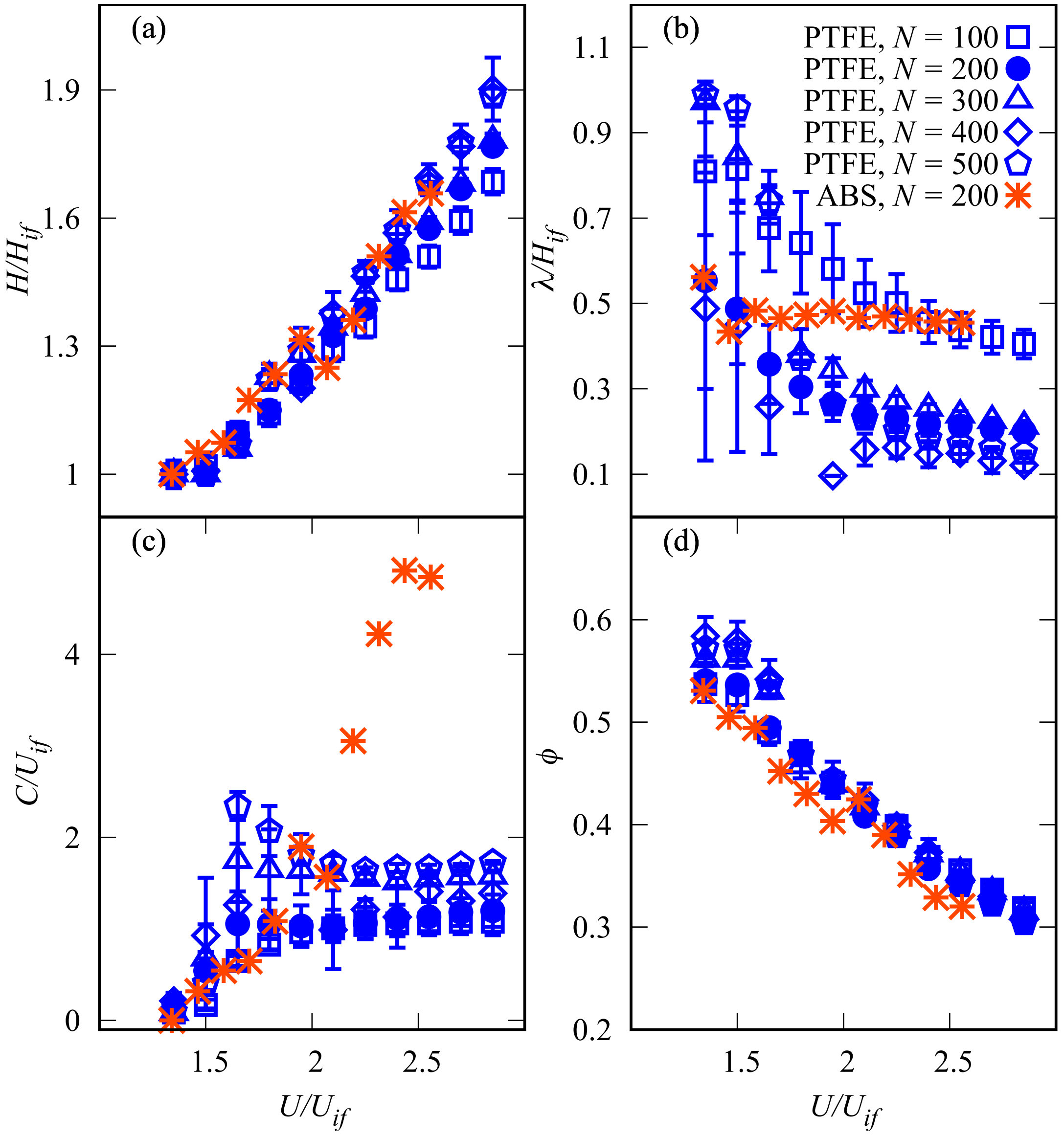

Figure S1. Dimensionless dynamics of the particle beds. (a) Dimensionless time averaged bed height, H/H_{if}, (b) plug height, λ/H_{if}, (c) plug celerity, C/U_{if} and (d) packing concentration, ϕ, as a function of the upwards mean dimensionless water velocity, U/U_{if}. H_{if} is the bed height and U_{if} the water velocity at incipient conditions. Errorbars represent standard deviations of five time average measuarens for each quantity.

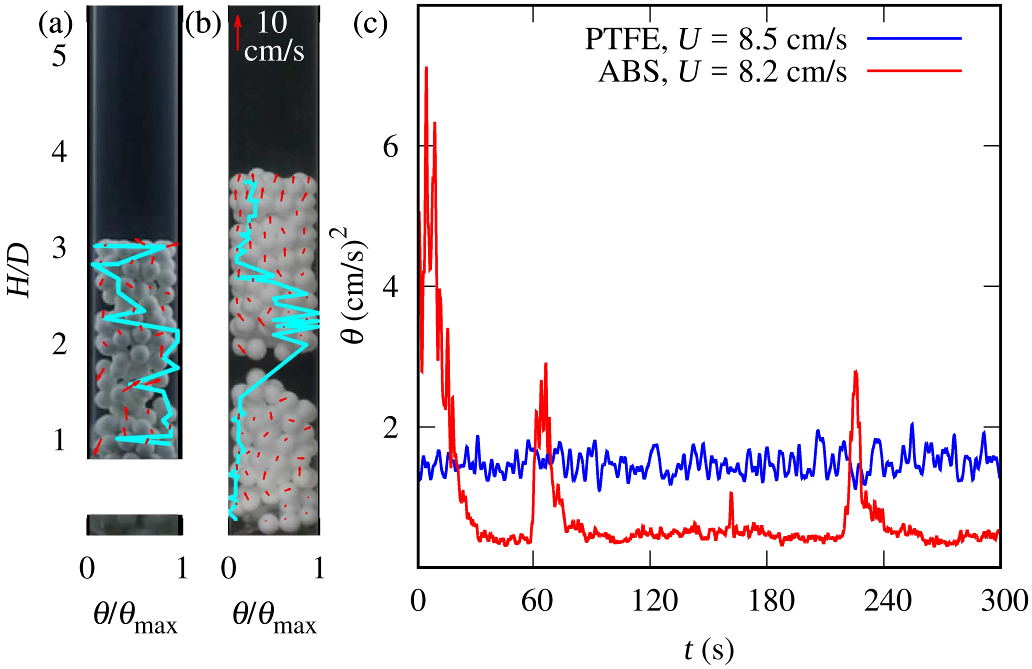

Figure S2. Snapshots of particle velocities and normalized local granular temperature θ/θ_{max} (a) for ABS with $N = 200$ and $U = 8.2$ cm/s and (b) for PTFE with $N = 200$ and $U = 8.5$ cm/s. The cyan lines represent the dimensionless local granular θ/θ_{max} of the granular temperature, where θ_{max} is the local maximum temperature. The red arrows represent the particles velocity, a scale is displayed at the top of the snapshot. (c) Evolution of global granular temperature θ for PTFE and ABS
